# Supplementary figures and images for: Widespread misidentification of scanning electron microscope instruments in the peer-reviewed materials science and engineering literature
Source: PLoS One. 2025 Jul 17;20(7):e0326754. doi: 10.1371/journal.pone.0326754 (PMC12270153; doi:10.1371/journal.pone.0326754)

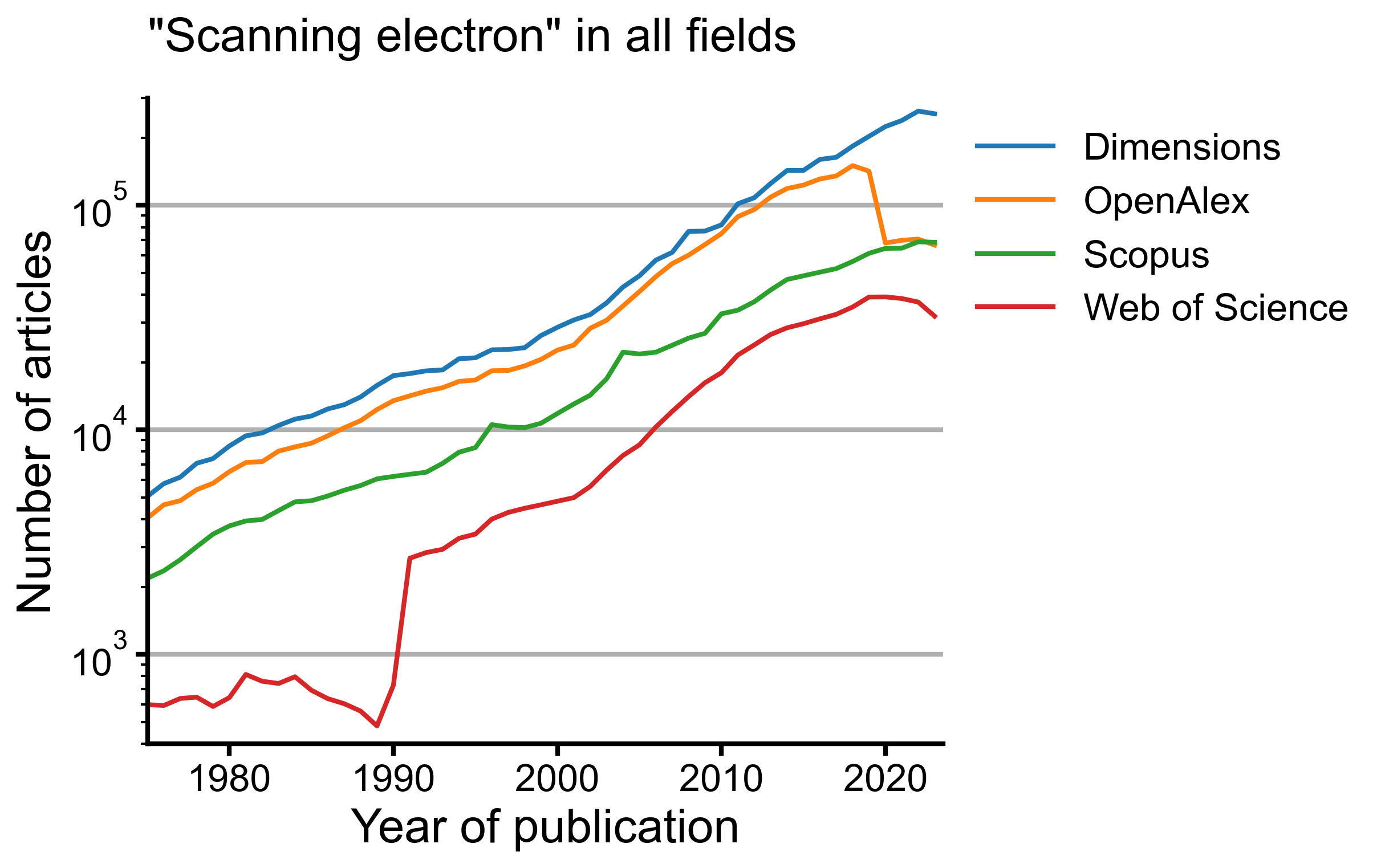

Supplement: S1 Fig — Yearly trends were obtained from each listed literature aggregator with a search of “Scanning electron" in all fields on January 24, 2024. (TIFF) [file pone.0326754.s001.tiff]

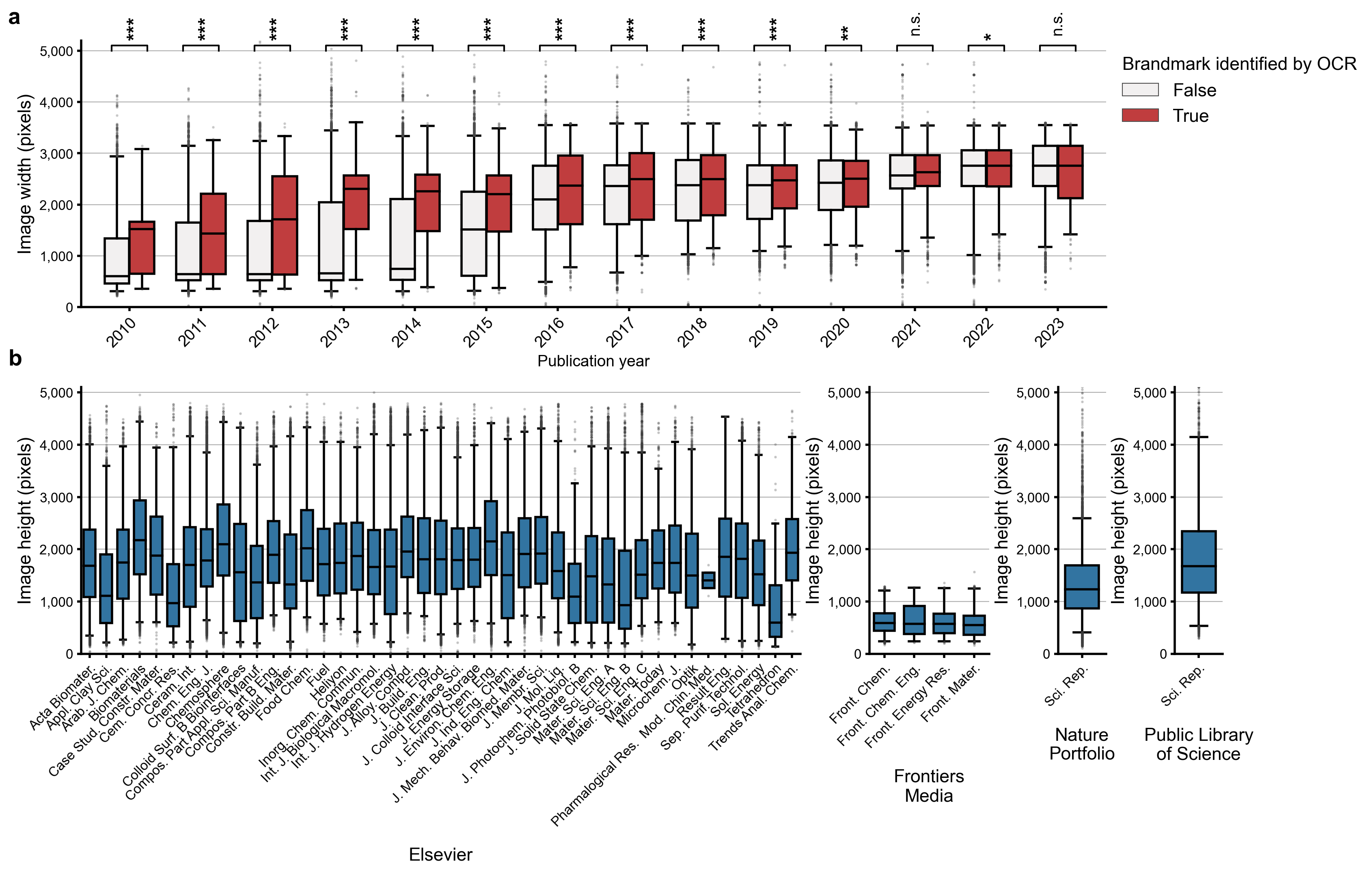

Supplement: S2 Fig — a, Images in which OCR was able to extract brandmarks (red boxes) were initially larger (in terms of image width) than images in which no brandmarks were identified (white boxes), but this difference is negligible in recent years. b, Image size (in terms of image height) varied considerably across publishers and within Elsevier’s portfolio. Center line shows median, boxes show inter-quartile range, whiskers show 2.5th percentile and 97.5th percentile. (TIFF) [file pone.0326754.s002.tiff]

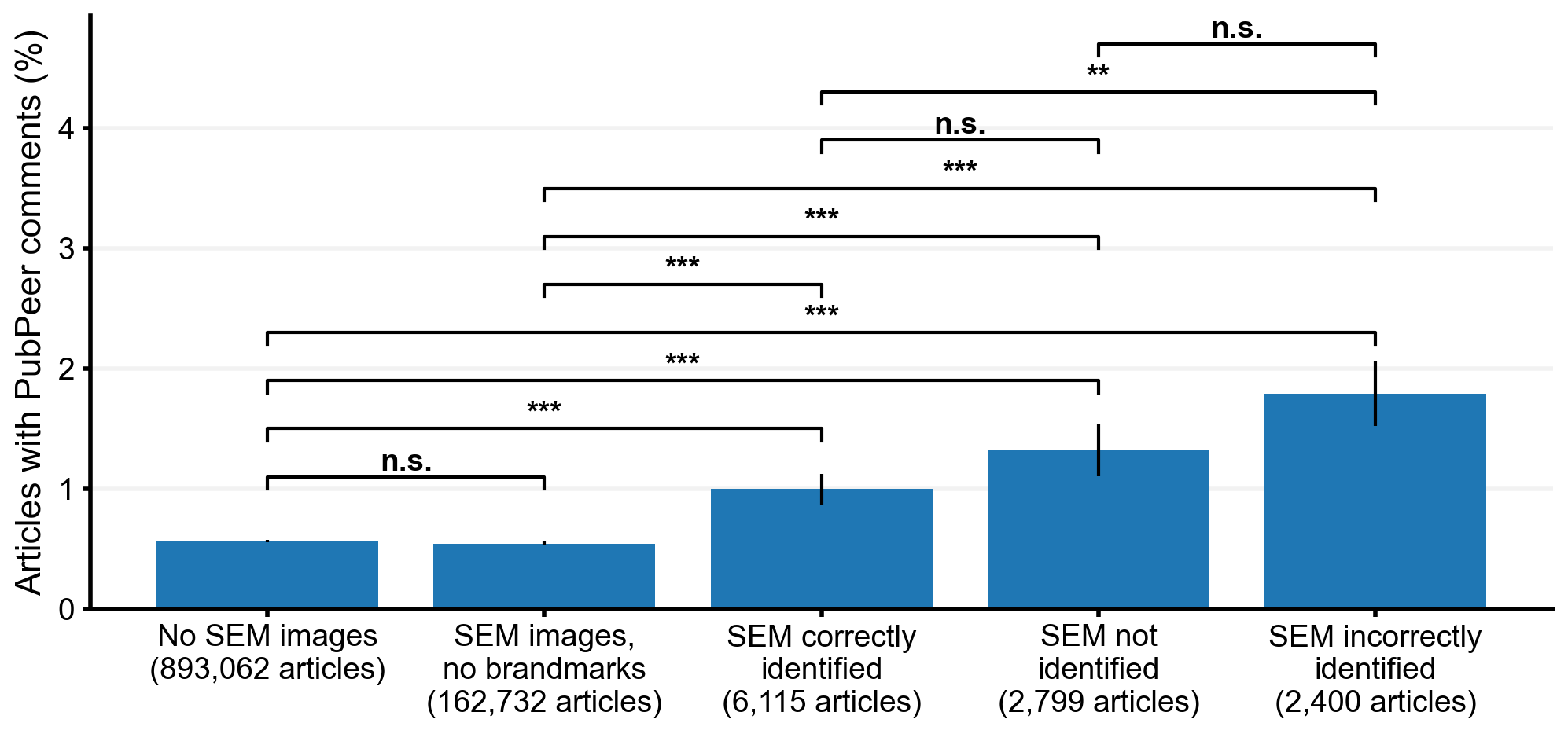

Supplement: S3 Fig — Pre-existing Pubpeer comments were more frequently found on articles with misidentified SEM instruments than other MSE articles. Errors bars show ±1 standard error of the proportion. n.s. = p > 0.05, * = p < 0.05, ** = p < 0.01 and *** = p < 0.001 by two-sided Z-test of proportions. (TIFF) [file pone.0326754.s003.tiff]

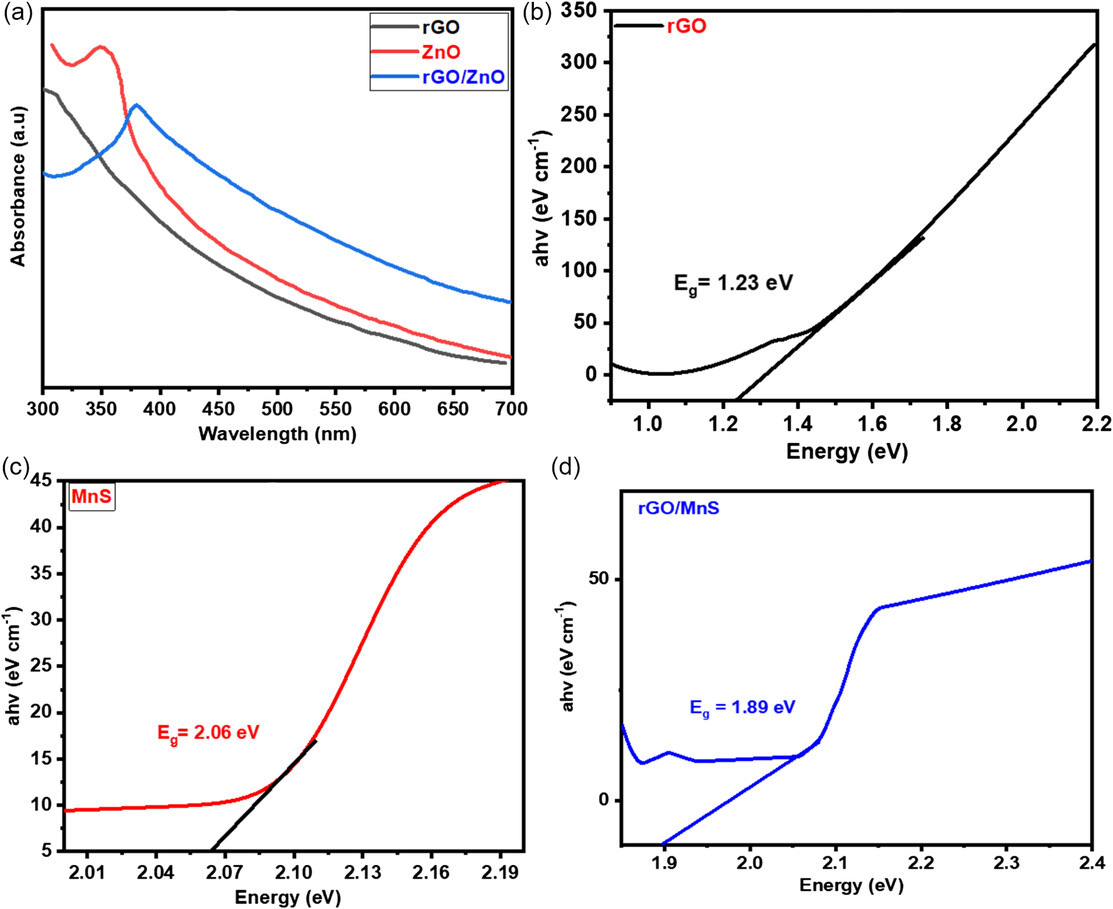

Supplement: S4 Fig — First, the Tauc plots shown for the three materials to not seem to resemble their corresponding absorbance spectra shown in the top left plot. Second, all three Tauc plots have a y-axis labeled “αhν (eV cm−1)”, which corresponds neither to an indirect ((αhν) 12) nor to a direct ((αhν) 2) allowed transition. Third, the linear fits in the Tauc plots for MnS (lower left) and rGO/MnS (lower right) are arbitrarily applied to the elbow of the curve and not the linear portion. Finally, in all three Tauc plots, the band gap energy Eg is evaluated at the lower limit of the y-axis instead of at y = 0. This final error is the specific error for which we annotated our sample of articles with both SEM images and Tauc plots. (TIFF) [file pone.0326754.s004.tiff]

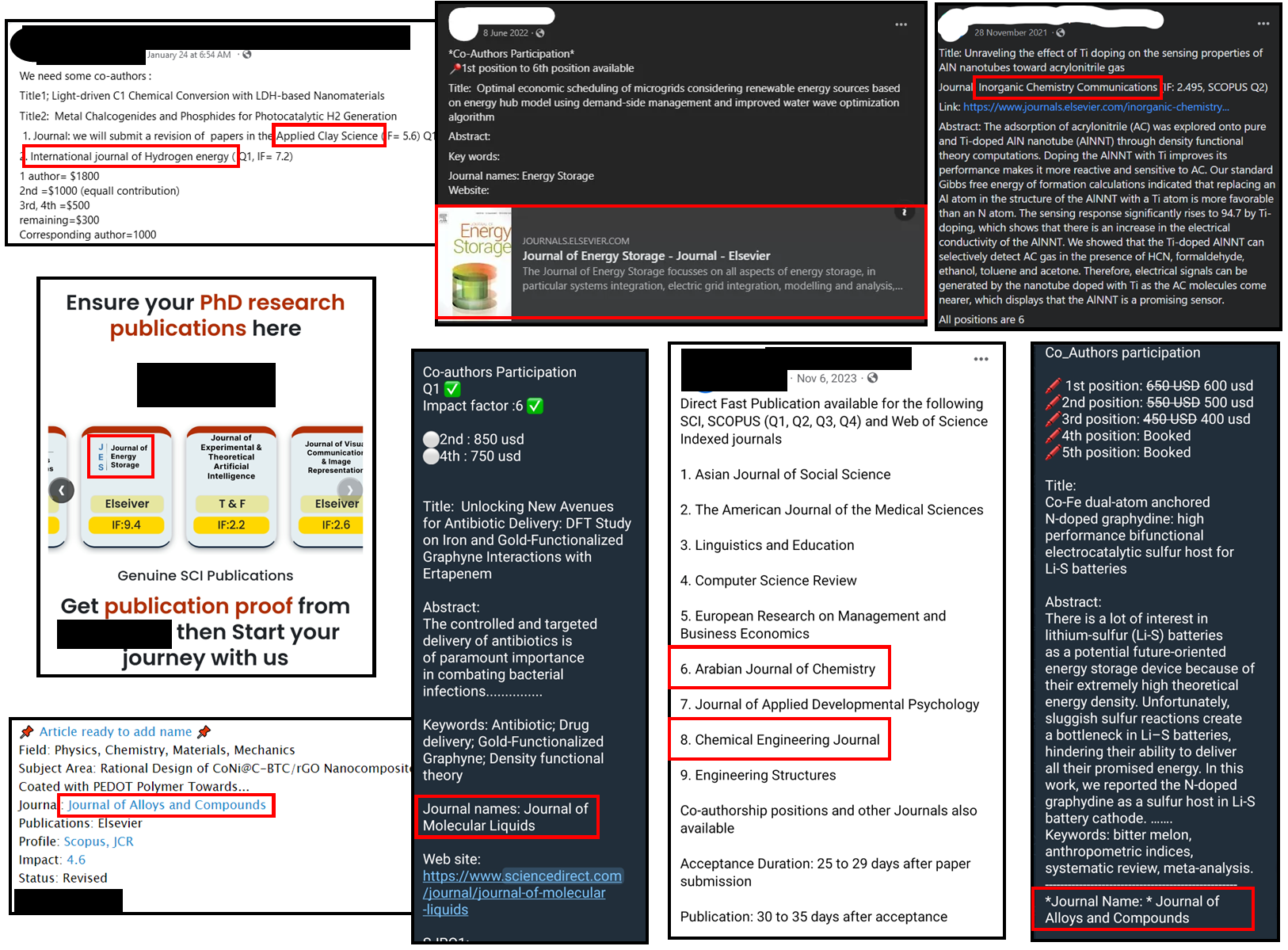

Supplement: S5 Fig — The names of journals that we surveyed are highlighted in red. Advertisements were found on Facebook, Telegram, WhatsApp and paper mill websites. (TIFF) [file pone.0326754.s005.tiff]
