# Supplementary material for: Widespread misidentification of scanning electron microscope instruments in the peer-reviewed materials science and engineering literature
Source: PLoS One. 2025 Jul 17;20(7):e0326754. doi: 10.1371/journal.pone.0326754 (PMC12270153; doi:10.1371/journal.pone.0326754)
Supplement: Attachment — Submitted filename: response_to_reviewers.pdf [file pone.0326754.s015.pdf]

Response to reviewers

Response:

We have provided a point-by-point response to the reviewer's comments below. We also provide a version of the manuscript with tracked changes from the last version. In addition to these comments, we identified two typos: in two instances, the total number of articles published in the 50 journals were surveyed was written as '1,067,102'. These have now been corrected to read '1,067,108'.

Reviewer #1:

The paper explores the reliability of materials science and engineering (MSE) research by analyzing the misidentification of scanning electron microscope (SEM) instruments in published articles. A semi-automated approach was developed to scan figures for SEM metadata and compare it with the instrument identified in the text. The analysis, which covered over a million articles published since 2010, revealed that 21.2% contained mismatched SEM metadata, while 24.7% did not report some of the instruments used. I appreciate the great efforts made by the authors. Through their analysis, the authors find the potential risks posed to MSE literature due to SEM misidentification, suggesting that these discrepancies may point to broader concerns such as systematic fraud and the presence of paper mills. Furthermore, articles with SEM misidentifications were more frequently associated with prior observations of improprieties recorded on post-publication peer review platforms. The authros also examined trends in authorship and institutional affiliations, noting that certain regions and institutions had a disproportionately high occurrence of SEM misidentification. Overall, the merits of the paper is sound and the paper is worth to be published. I believe this paper is crucial in raising awareness about the issue of SEM misidentification and its broader implications for research integrity. By bringing this problem to light, I hope to encourage researchers, reviewers, and editors to place greater emphasis on accurately reporting SEM instruments. Beyond just addressing SEM misidentification, I see this issue as a gateway to uncovering larger systemic problems in scientific publishing, such as research misconduct and the operation of paper mills. This work could inspire further investigations into the reliability of published studies and lead to the development of better tools for identifying problematic research. The semi-automated detection method proposed here has the potential to be implemented by journals and publishers, strengthening peer review processes and improving overall research quality.

Response:

We thank the reviewer for their positive evaluation of our manuscript.

Reviewer #2:

This manuscript presents a systematic investigation into the misidentification of scanning electron microscope (SEM) instruments in the materials science and engineering (MSE) literature. The

authors employ a semi-automated pipeline to analyze over a million articles, identifying significant discrepancies between reported SEM metadata in text and images. The study highlights critical issues in scientific reporting and suggests broader implications for research integrity. The work is well-structured, methodologically rigorous, and addresses an important gap in the literature. However, some areas require clarification and further discussion. Major comments The high false negative rate (71.1% of PubPeer-identified cases were missed) suggests limitations in the OCR-based approach. The authors should discuss potential improvements, such as alternative OCR tools or manual supplementation, to increase sensitivity.

Response:

> We thank the reviewer for this suggestion. We have added the following text to the Limitations section: "Improvements on this method to improve sensitivity might involve using a lightweight convolutional neural network, vision transformer or other vision model to identify figures containing SEM images with metadata banners, which could then be manually inspected and annotated instead of relying on OCR to discern brandmarks."

The reliance on high-resolution images for metadata extraction may bias the results toward recent publications or specific publishers. The authors should further discuss how this bias might affect the generalizability of their findings.

Response:

> We thank the reviewer for this suggestion. The first paragraph of our Limitations section now reads: "Our analysis is not without limitations. First, the size of image files for figures across the scientific literature increased substantially over the time period surveyed (**Figs. 2a** and **S2**). As a result, brandmarks are likely more readily identifiable via OCR in more recent articles than in older ones. Thus, we cannot conclusively determine whether, as suggested by **Fig. 4a**, the problem has become worse in recent years. Similarly, image sizes varied considerably across journals and publishers, which may mislead comparative analysis of SEM misidentification rates."

The exclusion of supplementary materials from the analysis is a notable limitation. The authors should justify this decision or address how supplementary data might alter their conclusions.

Response:

> We thank the reviewer for this suggestion. We now note the following in the Limitations section: "We arrived at the decision to exlcude supplementary materials because journals and publisher application programming interfaces (APIs) did not make supplementary material available in a standardized format, unlike the main text and main text figures. Processing information from supplementary materials could recover more instances of SEM misidentification and might reclassify some cases where we found no identification of SEM instruments. However, manual inspection of articles (including inspection of supplementary materials) labeled as having SEM misidentification only yielded additional context in a minority of cases (**Fig. 5a**). Thus, inclusion of supplementary materials is unlikely to alter false discovery rates."

While the study correlates SEM misidentification with other errors (e.g., Tauc plot mistakes), it does not establish causality. The authors should clarify whether these errors are symptomatic of broader issues (e.g., paper mills) or isolated oversights.

Response:

> We thank the reviewer for this suggestion. We have added the following to the Discussion section: “We also find that articles with SEM misidentification are more likely to have existing observations of improprieties made on post-publication peer review site PubPeer than other MSE articles and that a subset of these articles within the subfield of electrochemistry are more likely to incorrectly estimate the optical band gap if the article features SEM misidentification. Although this analysis is correlative in nature, these findings suggests that SEM misidentification may be a tractable signature for flagging problematic MSE articles.”

Minor Comments Some figures (e.g., Figure 2) are dense and could be simplified for better readability. Consider breaking complex figures into subpanels or providing clearer labels.

Response:

> We thank the reviewer for this suggestion. We have revised the caption of Figure 2 for clarity and accuracy: “**Comparison of the sizes of images, in terms of image file height in pixels, from which we were able to extract brandmarks using optical character recognition (OCR) (red boxes) versus images for which we were not (white boxes)**. **a,** For all years, image files for which we were able to extract brandmarks were larger. **b,** Comparison across 4 publishers of the sizes of images for which we were able to extract brandmarks (red boxes) versus images for which we were not (white boxes). Except studies published by the Public Library of Science, images for which we were able to extract brandmarks were significantly larger. Center line shows median, boxes show inter-quartile range, whiskers show 2.5th percentile and 97.5th percentile. n.s. = $p > 0.05$, * = $p < 0.05$, ** = $p < 0.01$ and *** = $p < 0.001$ by two-sided Mann-Whitney U test. Additional analysis shown in **Fig. S2**.”

The term ”problematic articles” is used frequently but could be more precisely defined (e.g., ”articles with SEM misidentification”).

Response:

> On the reviewer’s suggestion, we have replaced all instances of the term ”problematic articles” with ”articles with SEM misidentification”.

Ensure all references are up-to-date, particularly for tools like Tesseract and OpenAlex, which may have newer versions or related studies.

Response:

> All references are up-to-date.

Overall Recommendation This manuscript makes a significant contribution to the literature on research integrity in MSE. The methodological approach is robust, and the findings are compelling. However, addressing the limitations and incorporating the suggested improvements would strengthen the manuscript further.

Response:

We thank the reviewer for their feedback and their positive evaluation of our manuscript.

Reviewer #3:

The paper by Reese A.K. Anderson, Jeonghyun Moon, Spencer S. Hong, and Luis A. Nunes Amaral, proposed for publication in PLOS ONE, addresses a critical issue in quantitative science: the reproducibility of results. This problem is well-documented in biomedicine and social sciences, where large-scale replication studies have frequently failed to confirm reported findings. However, it has not been extensively studied in the physical-chemical sciences and engineering, despite high-profile retractions in areas such as room-temperature superconductivity, organic electronic devices, and Majorana fermions. With the exponential increase in scientific publications, there is growing interest in developing automated or semi-automated methods to identify erroneous or counterfactual claims in research articles. Publishers are beginning to integrate these methods into their protocols to detect unreliable content in unpublished manuscripts and combat automated mass production of scientific papers. To address this issue, the authors focus on the use of scanning electron microscopy (SEM) in materials science and engineering (MSE), a widely adopted technique for characterizing materials and their properties. SEM images typically include metadata banners providing details such as the manufacturer, instrument model, and imaging parameters. The study was inspired by reports on the post-publication peer review platform PubPeer, where users observed that metadata from SEM images in many articles did not correspond to the information provided in the text. These discrepancies were often accompanied by other inconsistencies. To investigate this issue, the authors developed a semi-automated method to detect SEM misidentification and applied it to over 1,000,000 articles published in 50 journals from four different publishers, with impact factors ranging from 2.1 to 24.2. Due to publisher restrictions on large-scale downloads, the study primarily analyzes articles from Elsevier, which has a more flexible data-mining policy. The developed method proved robust against false positives through manual verification, allowing the authors to identify patterns of misattributed SEM brandmarks and models. One example includes systematic errors in the calculation of the optical band gap using the Tauc plot method from electrochemical measurements. The study found that articles including both SEM images and Tauc plot were more likely to miscalculate the band gap if the SEM metadata was misreported. Additionally, recurring trends were identified based on the countries of origin of articles with incorrect SEM identification. The paper is well-written, with a clear discussion of the methodology and its limitations, and addresses a topic of broad interest to the scientific community. Thus, I consider it suitable for publication in PLOS ONE.

Response:

We thank the reviewer for their positive evaluation of our manuscript.

However, I would appreciate further clarification on the following points: 1. Selection of initial dataset: The authors state that they begin with an initial dataset of 1,067,108 articles. However, as indicated in Figure 3, only 16% of these contain SEM-related terms in figure captions. This suggests that the majority do not use SEM as a characterization method or include SEM images in the main text or supplementary materials. Merely mentioning ”scanning microscopy” does not necessarily imply the presence of SEM images. If the initial dataset is meant to highlight the technique's relevance, this should be made clearer. Otherwise, considering the full dataset skews the perceived percentage of misreporting.

Response:

> We thank the reviewer for this feedback. We'd like to clarify that that the initial dataset of 1,067,108 articles is only a starting point, from which we use criteria like "scanning electron" in a figure caption to identify articles that do indeed use SEM and contain SEM images. In our manuscript, we never use 1,067,108 as a denominator to determine rates of SEM misidentification. To allay this concern further, we manually inspected a random subset of 300 image files recovered using our string searching criteria. All 300 did indeed contain SEM images, suggesting that our criteria were sufficient for identifying studies that used SEM with high confidence.

2. Definition of ”resolution”: The term ”resolution” in the article is somewhat ambiguous, particularly in relation to the presence or absence of metadata banners in SEM images. Typically, resolution refers to the number of pixels in height and width. However, the authors focus solely on image height, noting that when it is too low, experimental metadata cannot be recovered. Often, when height is reduced, the banner is intentionally cropped—likely because SEM software does not allow users to adjust font size, making the metadata unreadable in publication formats. If this is the case, further clarification is needed on how cropped images are categorized.

Response:

> We thank the reviewer for this feedback. We'd like to make the following clarifications:
>
> 1) When downloading the image files of figures in the articles we examine, we only have access to metadata concerning the height and width of an image in pixels, not its print size, display size or resolution (as would be expressed in a unit like dots per inch). Although we have used this as a proxy for image resolution, the reviewer is correct that the two are not equivalent. We have revised our description of image size throughout the manuscript to more accurately reflect this.
>
> 2) After downloading image files, we perform no additional processing before performing OCR (e.g., panel segmentation, cropping, etc.). Thus, OCR is performed on an entire figure simultaneously, not just on portions containing SEM images. While smaller (i.e., shorter) images may appear so because the metadata banner has been cropped out, this is more likely a reflection of the size of the uploaded figure than the processing of SEM images contained within the figure. A large figure with an SEM image featuring an intact metadata banner may still have unrecoverable brandmarks because the SEM image is only one panel within the figure and the relevant portion

of the image is too small to recover text. We note that although figures have likely become more dense with subfigures and panels over time (see Lee et al. 10.1109/TBDATA.2017.2689038), implying that SEM images take up a smaller proportion of figures' areas now than in the past, we find that recovery of brandmarks increases over time. Thus, we conclude that image height is a suitable proxy for the size of SEM images within a figure.

3. Citation trends of misidentified SEM articles: According to the "Systemic Characteristics" section, articles with misidentified SEM metadata receive more citations on average than those with correct information. This is an intriguing observation. Could this be due to the prevalence of open-access journals, where articles with poor experimental details might be more frequently cited? Alternatively, could this indicate another form of misconduct related to citation practices? I would be interested in the authors' perspective on this correlation.

Response:

We share the reviewer's enthusiasm for this question. Any of these possibilities are plausible, but we lack sufficient data to test these hypotheses with appropriate control for confounding factors. Future work might consider how the presence of conspicuous errors (e.g., SEM misidentification, nucleotide reagent misreporting, tortured phrases, etc.) affects citation reception in the presence of appropriate controls for confounding factors like time of publication, journal-level metrics, author reputation, discipline, country of origin, methodology, etc.

4. Impact factor and SEM reporting: The study finds that lower-impact-factor journals are more likely to contain incorrect or incomplete information, which aligns with expectations. However, I was surprised to see in Table S1 that for Materials Today—the highest-impact journal analyzed—only 2 out of 474 articles containing SEM images included brandmarks, and only one of these was correctly reported. My assumption (and hope) is that the remaining 472 articles included correct SEM information elsewhere, even if it was not retrievable from metadata. The authors discuss the method's limitations, but in their conclusions, they suggest that these limitations inherently lead to an increased occurrence of misreported SEM data. This statement should be made with more caution.

Response:

We thank the reviewer for their feedback. We'd like to make the following clarifications:

1) We deliberately do not make any statements about whether journals with lower impact factors are more likely to contain inaccurate or incomplete information. We believe that our assembled dataset is insufficient to test this robustly because it is not a representative sample of the MSE literature and because of the high false negative rate of our pipeline.

2) When metadata banners are not included in SEM images, rates of SEM misidentification are inherently unknowable from the information presented in the article alone. Without metadata banners, it is impossible to determine which instrument was used and compare that information to the instrument that is claimed in the text. As we note

in the Data and Methods section, "SEM instrument misidentification is only detectable where metadata banners are visible in the published figures. In the vast majority of materials science articles where metadata banners are not visible, rates of instrument misidentification are entirely unknowable".

3) Informal discussions among colleagues suggest that certain high impact journals (e.g., *Nature*, *Science*, *Materials Today*) have more stringent stylistic guidelines that lead to the removal of SEM metadata banners from published figures.

Overall, the study presents a valuable contribution to the discussion of research integrity and reproducibility in materials science. Addressing the points raised above would further strengthen the clarity and impact of the findings. I also suggest to review the following:
Title. The use of acronyms in the titles should be avoided even if

Response:

We have edited our title to remove the abbreviation of "scanning electron microscope".

Figure 1. There is a typo in the text (extant).

Response:

This is not a typo; 'extant' means 'currently or actually existing' according to Merriam-Webster. To avoid confusion, we have removed this word.

Figure 6. In contrast to previous caption, here after any entry a, b, a minuscule letter is used. Revise the uniformity of notation all over the manuscript.

Response:

We thank the reviewer for spotting this formatting error. We have revised this figure caption and the figures in the supplement for consistency in style.

Figure S4. The first phrase of the caption is not clear (no verb).

Response:

We are not sure of the source of confusion for this phrasing. We have appended the article 'an' to the start of the phrase in case this helps understanding.

Reference. The reference style is not uniform across all the entries, and for many of the items the information is not complete (as it happens in mass-produced papers…). A full stop should be included at the end of each item entry.

Response:

Our references are formatting in the Vancouver style as specified in the *PLOS ONE* submission guidelines. We would welcome additional clarification from the reviewer on what information is missing. Should the manuscript be accepted, we will ensure that references are compliant during the copy-editing process.

Reviewer #4:

The manuscript presents the differences between the make and model of the SEM mentioned in the text and the data obtained from the image. The study made an investigation of a large number of papers on this subject by a semi-automated approach. Although the results of the study are valuable, I have concerns that some cases require evidence beyond allegations and that these cases may have legal consequences. Therefore, I would like to inform you that the allegations in Figure S5 have the potential to lead to legal proceedings.

Response:

> We thank the reviewer for their positive evaluation of our manuscript and appreciate their concern for the possibility of legal trouble as a result of its publication. We consider this possibility unlikely. We have redacted all identifying information shown in Figure S5 and do not name any individuals and organizations in our manuscript. Further, many studies have been published concerning systematic scientific fraud that make far more specific and actionable allegations (e.g., Abalkina 10.1002/leap.1574, Nielsen et al. 10.1016/j.jogoh.2024.102794, Gaumann and Veale 10.1080/13600834.2024.2352692), including by us (Richardson et al. 10.1007/s40979-025-00185-8). These investigations have not resulted in legal consequences for the authors nor did the publishers raise concerns prior to their publication. Should such concerns arise, we will work with the publisher to resolve them.

-In Figure 4b, the samples labeled as 'Mfr', it is not stated but it is understood that the abbreviation of the manufacturer. Also, in the whole text it was mentioned as both 'make' or 'manufacturer' and the abbreviation is not used. It would be better to use one of them in order to provide the uniformity throughout the text.

Response:

> We thank the reviewer for this suggestion. We have clarified the abbreviation in the caption of Figure 4 and have replaced all instances of 'make' with 'manufacturer' where appropriate.

-In heading of fig 5. b) 'For 30 fo the 43' please correct it.

Response:

> We thank the reviewer for spotting this typo and have corrected it.

-In line 240 'The five most common authors of articles with misidentified SEMs authored between 13 and 42 problematic articles, respectively.' What does the respectively correspond?

Response:

> We thank the reviewer for spotting this typo and have corrected it.

-In line 289 'The presence of repeated authorship (Fig. 7d)' There is no figure 7d.

Response:

We thank the reviewer for spotting this typo and have corrected it. The correct reference is to Figure 7b.

- The resolution of the graphs is so low.

Response:

This is an artifact of *PLOS ONE*'s submission system. We have supplied original, high-resolution figures to the publisher and will ensure they appear that way in the final publication.

Reviewer #5:

This is a solid and detailed work about a semi-automatic pipeline to identify SEM instrument information issues in published research papers. The authors focused on the materials science field and examined more than 1 million published works from 50 journals with a diversified set of impact factors. The authors made several significant claims supported by clearly identified numbers. I especially appreciate the: (1) well-prepared figures, (2) well-organized sections, and (3) comprehensive supporting figures and tables.

Response:

We thank the reviewer for their positive evaluation of our manuscript.

I support the publication of this work, after some minor revisions are made, including:
1. In Figure 2, image height is used as the indicator of the image resolution. I don't think this is strictly correct, since usually the image resolution is represented by both height and width pixel counts. I think it would be better if the y axis can be updated to a unit like pixels per inch to indicate the image resolution.

Response:

We thank the reviewer for this feedback. When downloading the image files of figures in the articles we examine, we only have access to metadata concerning the height and width of an image in pixels, not its print size, display size or resolution (as would be expressed in a unit like dots per inch). Although we have used this as a proxy for image resolution, the reviewer is correct that the two are not equivalent. We have revised our description of image size throughout the manuscript to more accurately reflect this.

2. Line 162: the claim about the underestimation of global misidentification rate is not necessarily true. The authors should (1) provide an estimation of the misidentification rate of the OCR-unreadable articles and (2) compare it to the OCR-readable misidentification rate. Only then can the authors make an estimated claim of the misidentification rate shift.

Response:

We thank the reviewer for this feedback and agree with their assessment. We would like to clarify that we meant because our method fails to capture many instances where

metadata banners are readable by eye but not by OCR, we may underestimate the global rate of SEM misidentification *among all articles containing SEM images*. We have revised this statement to this effect: "This suggests that our approach may underestimate the global rate of recognizable SEM instrument misidentification among articles containing SEM images, especially for journals with low standards for sizes of images published as figures."

3. Paragraph starting from line 193: the authors found the proposed pipeline has a high false negative rate (showed in Figure 5c). How does this observation affect the number of papers with incorrectly identified SEM instruments in Figure 4a? Since this false negative rate is high (71.1%), the authors should consider duplicating some of the discussions from line 278 in this paragraph for easier-accessible information for the reader when reading this part.

Response:

We thank the reviewer for this suggestion. We have added the following text to this section: "This suggests that SEM misidentification could be more than twice as frequent as what we detect with our pipeline."

4. Other minor issues:
4.1 Figure 2b, include the full form of "OCR" in the legend or in the figure caption.

Response:

We thank the reviewer for this suggestion. We have revised the figure caption header to read: "Comparison of the sizes of images, in terms of image file height in pixels, from which we were able to extract brandmarks using optical character recognition (OCR) (red boxes) versus images for which we were not (white boxes)."

4.2 In the supplementary spreadsheet, fix the caption for Table S4 (referred as Table S3) and Table S5 (referred as Table 5). The font size can also be consolidated across the sheets.

Response:

We thank the reviewer for spotting these typos. We have fixed them and have ensured a consistent font size across all sheets.

4.3 Figure 5b caption: "For 30 fo the 43" should be corrected.

Response:

We thank the reviewer for spotting this typo and have corrected it.

Reviewer #6:

Very solid piece of work! It raises very important issues about reproducibility and validity of reported results and suggests options to analyse and detect most common faults that prevent FAIR principles.

Response:

We thank the reviewer for their positive evaluation of our manuscript.
