# Supplementary material for: Widespread misidentification of scanning electron microscope instruments in the peer-reviewed materials science and engineering literature
Source: PLoS One. 2025 Jul 17;20(7):e0326754. doi: 10.1371/journal.pone.0326754 (PMC12270153; doi:10.1371/journal.pone.0326754)
Supplement: Attachment — Submitted filename: PLOSONE_review.docx [file pone.0326754.s014.docx]

The paper by Reese A.K. Anderson, Jeonghyun Moon, Spencer S. Hong, and Luis A. Nunes Amaral, proposed for publication in PLOS ONE, addresses a critical issue in quantitative science: the reproducibility of results. This problem is well-documented in biomedicine and social sciences, where large-scale replication studies have frequently failed to confirm reported findings. However, it has not been extensively studied in the physical-chemical sciences and engineering, despite high-profile retractions in areas such as room-temperature superconductivity, organic electronic devices, and Majorana fermions. With the exponential increase in scientific publications, there is growing interest in developing automated or semi-automated methods to identify erroneous or counterfactual claims in research articles. Publishers are beginning to integrate these methods into their protocols to detect unreliable content in unpublished manuscripts and combat automated mass production of scientific papers.

To address this issue, the authors focus on the use of scanning electron microscopy (SEM) in materials science and engineering (MSE), a widely adopted technique for characterizing materials and their properties. SEM images typically include metadata banners providing details such as the manufacturer, instrument model, and imaging parameters. The study was inspired by reports on the post-publication peer review platform PubPeer, where users observed that metadata from SEM images in many articles did not correspond to the information provided in the text. These discrepancies were often accompanied by other inconsistencies. To investigate this issue, the authors developed a semi-automated method to detect SEM misidentification and applied it to over 1,000,000 articles published in 50 journals from four different publishers, with impact factors ranging from 2.1 to 24.2.

Due to publisher restrictions on large-scale downloads, the study primarily analyzes articles from Elsevier, which has a more flexible data-mining policy. The developed method proved robust against false positives through manual verification, allowing the authors to identify patterns of misattributed SEM brandmarks and models. One example includes systematic errors in the calculation of the optical band gap using the Tauc plot method from electrochemical measurements. The study found that articles including both SEM images and Tauc plot were more likely to miscalculate the band gap if the SEM metadata was misreported. Additionally, recurring trends were identified based on the countries of origin of articles with incorrect SEM identification.

The paper is well-written, with a clear discussion of the methodology and its limitations, and addresses a topic of broad interest to the scientific community. Thus, I consider it suitable for publication in PLOS ONE. However, I would appreciate further clarification on the following points:

1. **Selection of initial dataset:** The authors state that they begin with an initial dataset of 1,067,108 articles. However, as indicated in Figure 3, only 16% of these contain SEM-related terms in figure captions. This suggests that the majority do not use SEM as a characterization method or include SEM images in the main text or supplementary materials. Merely mentioning "scanning microscopy" does not necessarily imply the presence of SEM images. If the initial dataset is meant to highlight the technique's relevance, this should be made clearer. Otherwise, considering the full dataset skews the perceived percentage of misreporting.
2. **Definition of "resolution":** The term "resolution" in the article is somewhat ambiguous, particularly in relation to the presence or absence of metadata banners in SEM images. Typically, resolution refers to the number of pixels in height and width. However, the authors focus solely on image height, noting that when it is too low, experimental metadata cannot be recovered. Often, when height is reduced, the banner is intentionally cropped—likely because SEM software does not allow users to adjust font size, making the metadata unreadable in publication formats. If this is the case, further clarification is needed on how cropped images are categorized.
3. **Citation trends of misidentified SEM articles:** According to the "Systemic Characteristics" section, articles with misidentified SEM metadata receive more citations on average than those with correct information. This is an intriguing observation. Could this be due to the prevalence of open-access journals, where articles with poor experimental details might be more frequently cited? Alternatively, could this indicate another form of misconduct related to citation practices? I would be interested in the authors' perspective on this correlation.
4. **Impact factor and SEM reporting:** The study finds that lower-impact-factor journals are more likely to contain incorrect or incomplete information, which aligns with expectations. However, I was surprised to see in Table S1 that for Materials Today—the highest-impact journal analyzed—only 2 out of 474 articles containing SEM images included brandmarks, and only one of these was correctly reported. My assumption (and hope) is that the remaining 472 articles included correct SEM information elsewhere, even if it was not retrievable from metadata. The authors discuss the method’s limitations, but in their conclusions, they suggest that these limitations inherently lead to an increased occurrence of misreported SEM data. This statement should be made with more caution.

Overall, the study presents a valuable contribution to the discussion of research integrity and reproducibility in materials science. Addressing the points raised above would further strengthen the clarity and impact of the findings. I also suggest to review the following:

Title. The use of acronyms in the titles should be avoided even if

Figure 1. There is a typo in the text (extant).

Figure 6. In contrast to previous caption, here after any entry a, b, a minuscule letter is used. Revise the uniformity of notation all over the manuscript.

Figure S4. The first phrase of the caption is not clear (no verb).

Reference. The reference style is not uniform across all the entries, and for many of the items the information is not complete (as it happens in mass-produced papers…). A full stop should be included at the end of each item entry.
